# Supplementary material for: Seminoma and Embryonal Carcinoma Footprints Identified by Analysis of Integrated Genome-Wide Epigenetic and Expression Profiles of Germ Cell Cancer Cell Lines
Source: PLoS One. 2014 Jun 2;9(6):e98330. doi: 10.1371/journal.pone.0098330 (PMC4041891; doi:10.1371/journal.pone.0098330)
Supplement: File S1 — ZIP file containing DMRforPairs output for significant regions. Please start from the html files. (ZIP) [file pone.0098330.s008.zip › figures/5372.pdf]

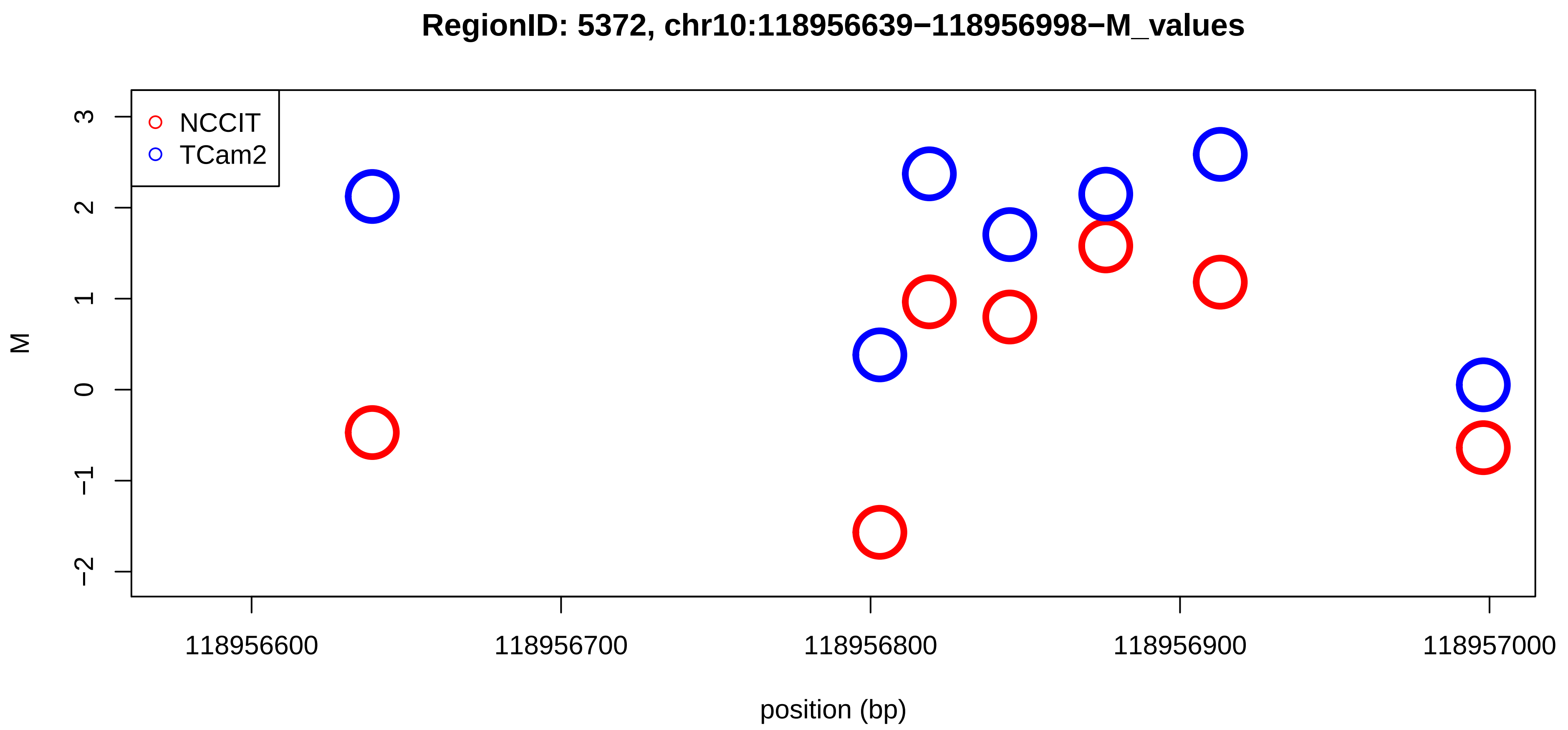

RegionID: 5372, chr10:118956639−118956998−M_values
NCCIT
TCam2
M
3
2
1
0
−1
−2
118956600
118956700
118956800
118956900
118957000
position (bp)

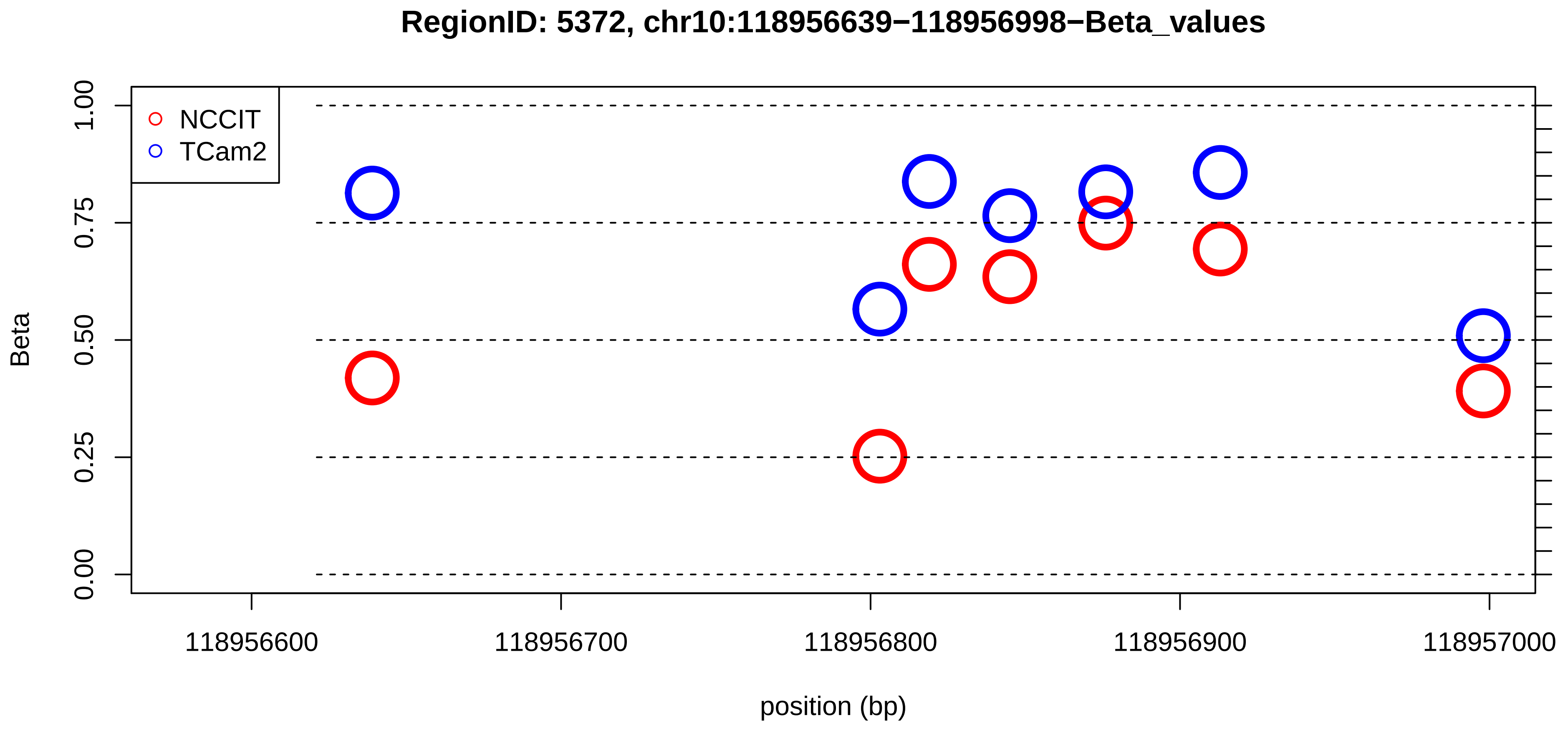

RegionID: 5372, chr10:118956639−118956998−Beta_values
NCCIT
TCam2
Beta
1.00
0.75
0.50
0.25
0.00
118956600
118956700
118956800
118956900
118957000
position (bp)
